# Efficacy of Wharton Jelly Mesenchymal Stromal Cells infusions in moderate to severe SARS-Cov-2 related acute respiratory distress syndrome: a phase 2a double-blind randomized controlled trial

**DOI:** 10.3389/fmed.2023.1224865

**Published:** 2023-08-29

**Authors:** Cécile Pochon, Caroline Laroye, Antoine Kimmoun, Loic Reppel, Adéle Dhuyser, Hélène Rousseau, Mélanie Gauthier, Nadine Petitpain, Jean-François Chabot, Simon Valentin, Marcelo de Carvalho Bittencourt, Michael Peres, Alice Aarnink, Véronique Decot, Danièle Bensoussan, Sébastien Gibot

**Affiliations:** ^1^CHRU-Nancy, Pediatric Onco-Hematology Department, Nancy, France; ^2^Team 6 IMoPA, UMR 7365 CNRS-UL, Université de Lorraine, Nancy, France; ^3^CHRU-Nancy, Unité de Thérapie Cellulaire et banque de tissus, Nancy, France; ^4^CHRU-Nancy, Service de Médecine Intensive et Réanimation, Hôpitaux de Brabois, Nancy, France; ^5^Université de Lorraine, Nancy, France; ^6^CHRU-Nancy, HLA and Histocompatibility Laboratory, Nancy, France; ^7^CHRU-Nancy, Département Méthodologie, Promotion, Investigation, Hôpitaux de Brabois, Nancy, France; ^8^CHRU-Nancy, Département de Pharmacovigilance, Hôpitaux de Brabois, Nancy, France; ^9^CHRU-Nancy, Pôle des Spécialités Médicales/Département de Pneumologie, Hôpitaux de Brabois, Nancy, France; ^10^CHRU-Nancy, Laboratoire d’immunologie, Hôpitaux de Brabois, Nancy, France; ^11^CHRU-Nancy, Service de Médecine Intensive et Réanimation, Hôpital Central, Nancy, France

**Keywords:** ARDS, COVID-19, mesenchymal stromal cells, intensive care, oxygenation

## Abstract

**Background:**

The COVID-19 pandemic caused a wave of acute respiratory distress syndrome (ARDS) with a high in-hospital mortality, especially in patients requiring invasive mechanical ventilation. Wharton Jelly-derived Mesenchymal Stromal Cells (WJ-MSCs) may counteract the pulmonary damage induced by the SARS-CoV-2 infection through pro-angiogenic effects, lung epithelial cell protection, and immunomodulation.

**Methods:**

In this randomized, double-blind, placebo-controlled phase 2a trial, adult patients receiving invasive mechanical ventilation for SARS-CoV-2 induced moderate or severe ARDS were assigned to receive 1 intravenous infusion of 1 × 10^6^ WJ-MSCs/kg or placebo within 48 h of invasive ventilation followed by 2 infusions of 0.5 × 10^6^ WJ-MSCs/kg or placebo over 5 days. The primary endpoint was the percentage of patients with a PaO_2_/FiO_2_ > 200 on day 10.

**Results:**

Thirty patients were included from November 2020 to May 2021, 15 in the WJ-MSC group and 15 in the placebo group. We did not find any significant difference in the PaO_2_/FiO_2_ ratio at day 10, with 18 and 15% of WJ-MSCs and placebo-treated patients reaching a ratio >200, respectively. Survival did not differ in the 2 groups with a 20% mortality rate at day 90. While we observed a higher number of ventilation-free days at 28 days in the WJ-MSC arm, this difference was not statistically significant (median of 11 (0–22) vs. 0 (0–18), *p* = 0.2). The infusions were well tolerated, with a low incidence of anti-HLA alloimmunization after 90 days.

**Conclusion:**

While treatment with WJ-MSCs appeared safe and feasible in patients with SARS-CoV2 moderate or severe ARDS in this phase 2a trial, the treatment was not associated with an increased percentage of patients with P/F > 200 at 10d, nor did 90 day mortality improve in the treated group.

**Clinical trial registration:**

https://beta.clinicaltrials.gov/study/NCT04625738, identifier NCT04625738.

## Introduction

In December 2019, the first cases of SARS-CoV-2 infection appeared in China. The phenomenon quickly became global and the pandemic settled permanently all around the world. Many treatments were tested with varying degrees of success. Among these, Mesenchymal Stem/stromal Cells (MSCs) appeared to be an attractive option.

These multipotent cells are characterized to be adherent to plastic, positive to mesenchymal markers CD73 CD90, and CD105, negative to hematopoietic markers HLA-DR, CD45, and CD34, and able to differentiate into osteocytes, chondrocytes, and adipocytes, according to the ISCT criteria (1). Found in many tissues, like bone marrow, adipose tissue, Wharton Jelly (WJ), and placenta, MSCs are of particular interest thanks to their immunomodulatory properties. They can modulate both innate and adaptive immunity by cell contact, secretion of cytokines, chemokines, growth factors, and release of extracellular vesicles as well as by mitochondrial transfer. Their action can be either «immunoprotective» or «immunosuppressive», depending on the inflammatory context and their Toll-Like Receptor (TLR) stimulation (2). Indeed, MSCs can inhibit lymphocyte proliferation, polarize pro-inflammatory macrophages (M1) into anti-inflammatory macrophages (M2), or inhibit B lymphocyte proliferation and immunoglobulin production in a pro-inflammatory context (3, 4). However, they can increase macrophage phagocytosis by mitochondrial transfer, promote NK cytotoxic functions, and T lymphocyte proliferation in an anergic cellular context (5–7).

Thus, their powerful immunomodulatory capacities make them attractive in several pathologies and notably for acute respiratory distress syndrome (ARDS) where inflammation and immune alterations are particularly prevalent. Pre-clinical studies have shown that MSC infusions in murine ARDS reduced pulmonary inflammation, improved alterations of immune metabolism, increased phagocytosis, and reduced alveolar apoptosis, contributing to survival improvement (8–10). In humans, the clinical trial “START” was one of the first to report on the use of MSCs during ARDS. The authors showed that administration of allogeneic MSCs derived from bone marrow appeared to be safe and well tolerated (11). Moreover, a recent meta-analysis of five clinical trials involving 455 COVID-19 patients (272 in the MSC group, 183 in the control group) suggested a potential efficacy of MSCs in this setting (12). Numerous trials have been carried out throughout the world to assess the impact of MSCs from different sources and their exosomes in COVID19 infection (13).

Here we report on the use of MSCs derived from umbilical cord WJ-MSCs during moderate to severe SARS-CoV-2-related ARDS in a phase 2a randomized controlled trial.

## Methods

### Study design and oversight

The MSC-COVID (Mesenchymal Stem Cells for COVID-19) trial is an investigator-initiated, randomized, sham infusion-controlled, parallel-group, double-blind, superiority clinical trial performed at the University Hospital of Nancy, France. The trial was conducted following the principles of the Declaration of Helsinki and the Good Clinical Practice guidelines of the International Council for Harmonization and was approved by the appropriate French ethics committee (Comité de Protection des Personnes Sud Mediterranee III, reference: EudraCT 2020-002772-12, acceptance on 17 November 2020). The trial was overseen by an external and independent data and safety monitoring board (DSMB). The MSC_COVID trial was registered with the number NCT04625738.^1^

### Participants

Patients admitted to two intensive care units were eligible to participate in the MSC-COVID trial if they were adults, had a positive RT-PCR for SARS-CoV-2 with moderate or severe ARDS according to the Berlin definition (14), and had been receiving invasive mechanical ventilation for less than 48 h. Written informed consent was obtained from the patient or his legal representative before enrollment. Exclusion criteria were: invasive mechanical ventilation >48 h, chronic respiratory disease under oxygen therapy, history of functional class III or IV pulmonary hypertension (WHO classification), extracorporeal membrane oxygenation treatment, immunosuppressive therapy, active solid tumor or in remission for less than 2 years, malignant hematological disease, asplenia, hematopoietic stem cells or organ transplantation, expected death within 24 h, a positive blood pregnancy test at inclusion, and participation in another interventional clinical trial.

### Randomization

Patients were randomly assigned in a 1:1 ratio to receive either WJ-MSCs plus standard of care (experimental group) or placebo plus standard of care (control group), as described below. Randomization was performed using a computer-generated allocation sequence, with permuted blocks of four and stratified according to the PaO_2_/FiO_2_ ratio at inclusion (≤100 or >100 mmHg). Patients and investigators were blinded.

### Treatments

In the experimental group, patients were assigned to receive three infusions of WJ-MSCs, in a solution of albumin 4% (40% of final volume), NaCl 0.9% (50% of final volume), and ACD (anticoagulant citrate dextrose) formula A (10% of final volume), with an interval of 2 days between two infusions. This treatment was administered intravenously for 10 minutes according to the following scheme: 1 × 10^6^ MSC/kg of body weight (with a maximum of 80 × 10^6^ MSC) at day 0 (or day 1) of inclusion, 0.5 × 10^6^ MSC/kg (with a maximum of 40 × 10^6^ MSC) at day 3 (or day 4) and then, at day 5 (or day 6). In the control group, patients were assigned to receive three infusions of placebo (albumin 4%, NaCl 0.9%, and ACD formula A, 75 to 100 mL) according to the same scheme. Experimental and control bags were strictly similar in order to maintain blinding.

The standard of care for COVID-19 was at the discretion of the physicians. Lung protective ventilation was applied to all patients. The use of corticosteroids, vasopressors, tocilizumab, and antibiotics was allowed.

### WJ-MSCs production

MSC production was performed in the Advanced Therapy Medicinal Product (ATMP) department of the cell therapy unit of Nancy Hospital. MSCs were isolated from Wharton’s jelly of umbilical cords, according to the explant method. The cells were qualified during production, before freezing, and upon thawing. The release of the cryopreserved batches was based on the following quality controls: infectious markers of the donor, cell viability, cell count, phenotype, karyotype, and microbiology. Additional quality controls were also performed to characterize MSCs: mixed lymphocyte reaction, clonogenicity, and telomerase activity (Supplementary data).

### Analysis of immune cells and inflammatory cytokines in peripheral blood

Immune cells were screened on days 1, 3, 5, 7, 10, 14, and 28 by flow cytometry for CD4+ T-cells, CD8+ T-cells, NK, Tregs cells (defined as CD4 + CD25 high CD127 low T cells), and myeloid-derived suppressive cells (MDSC, defined as HLA-DR^−^ lin^−^, CD33^+^ CD11b^+^, and CD14^+^ for monocytic MDSCs, CD15^+^ for granulocytic MDSCs, and CD14^−^CD15^−^ for precursor MDSCs).

Blood plasma was collected and frozen (i) just before the first infusion, and the day after the last infusion for the measurement of Angiopoietin 2, soluble receptor for advanced glycation end products (sRAGE), and (ii) on days 1, 3, 5, 7, 10, 14, and 28 for the measurement of Galectin-3, Galectin-9, interferon gamma (IFN-γ), tumor necrosis factor alpha (TNF-α), interleukines (IL) IL-1α, IL-1β, IL-10, vascular endothelial growth factor a (VEGF-A), interferon gamma induced protein 10 (IP10), and latency-associated peptide(LAP) by multiplex ELISA (Luminex xMAP technology, Luminex Corporation, Austin, United States) after completion of the trial.

### Analysis of viral load by SARS-CoV2 RT-PCR

Viral load of SARS-CoV2 was detected by semi-quantitative RT-PCR in tracheal or nasal aspirates on days 0, 7, 14, 21, and 28 (or the last day of hospitalization).

### Analysis of anti-HLA antibodies

Sera were collected on days 0, 28, and 90 in order to detect anti-HLA immunization. Thanks to Luminex xMAP technology, antibodies targeting class I and class II molecules were first screened using Labscreen Mixed Class I and II (OneLambda, Canoga Park, United States). In case of a positive screening, single antigen detections of the positive class were performed. An anti-HLA immunization towards MSCs was assessed if an antibody targeting MSCs’ HLA molecules (i.e., donor-specific antibody, DSA) was present with a mean intensity of fluorescence (MFI) above 1,000.

### Medium-term respiratory evaluation

Patients were followed up on day 90 with a clinical examination, chest computed tomography, spirometry, and 6 minute walk distance. Respiratory morbidity was graded as very severe when forced vital capacity (FVC) < 30% and/or need for oxygen and/or presence of fibrosis, severe when 30 < FVC < 50% or presence of reticulations, and moderate when 50 < FVC < 80% or presence of ground glass opacities.

### Outcomes and follow-up

The primary endpoint was the percentage of patients with a PaO_2_/FiO_2_ > 200 mmHg at day 10 of treatment (WJ-MSC or placebo).

Secondary endpoints were the PaO_2_/FiO_2_ evolution between the first day of infusion (day 0 or 1) and day 14, the number of 28 day ventilator, vasopressor, and extra-renal support free days, the difference in Sequential Organ Failure Assessment (SOFA) score between day 14 and day 0, 90 day all-cause mortality, ICU length of stay, respiratory morbidity at day 90, and RT-PCR SARS-CoV-2 positivity at day 7, 14, and 21.

Safety endpoints included any infusion-related toxicity (hypersensitivity reaction within 6 h of infusion), D-dimers elevation at day 10, acquisition of anti-HLA antibodies at day 28 and day 90, and the occurrence of thromboembolic events or infectious events within 90 days post-randomization.

### Statistical analysis

At the time of the study design, only data available were from Sengupta et al. study (14) showing that 80% of COVID-19 patients treated with exosomes derived from bone marrow MSC achieved a PaO_2_/FiO_2_ ratio >200 mmHg at day 3. We therefore hypothesized that the percentage of patients with a PaO_2_/FiO_2_ ratio >200 mmHg at day 10 would be 75 and 25% in the MSC and control groups, respectively. Based upon an *α* risk at 5% and a power of 80%, 14 patients were needed in each group.

The qualitative variables are presented as numbers and percentages, and the quantitative ones as median and range. Missing data were not imputed except for the main outcome.

Data were analyzed according to the intention-to-treat principle. Bivariate analyses were used to compare patients’ characteristics at inclusion between groups and to assess the treatment effect on outcomes.

The percentage of patients having a PaO_2_/FiO_2_ ratio >200 mmHg on day 10 was compared between groups using the Fisher exact test, as well as the percentage of patients with a positive RT-PCR SARS-CoV-2 at days 7, 14, and 21.

The median number of days alive and free of ventilator, extra-renal support, or vasopressor, and the ICU length of stay were compared between groups by using a Mann–Whitney test. The comparison between groups of the evolution of PaO_2_/FiO_2_ between day 0 and day 14 was performed by a repeated measures ANOVA model and the difference in SOFA score between day 14 and day 0 by a Wilcoxon signed rank test. LogRank tests were used to compare survival curves generated by the Kaplan–Meier method.

For comparison of adverse events between groups, a clustering patient effect was first tested by calculating the intraclass coefficient correlation (ICC). A one-level hierarchical logistic model was then used if a patient effect was identified (ICC > 0); otherwise, a logistic model was used.

A threshold of *p* = 0.05 for two-tailed tests was considered significant. Statistical analyses were performed using SAS version 9.4 software (SAS Institute Inc.).

## Results

### WJ-MSC production

All the cryopreserved MSC batches used complied with the specifications (see Supplementary Table S1). Viability of infused cells was 80.69 +/− 10.94%. The first infusion contained a mean of 0.80 +/− 0.16 × 10^6^ MSCs/kg (0.47 × 10^6^ to 1 × 10^6^ MSCs/kg). The second and third infusions contained a mean of 0.41 +/− 0.07 × 10^6^ MSCs/kg (0.22 × 10^6^ to 0.5 × 10^6^ MSCs/kg).

### Patients

**Figure 1:**
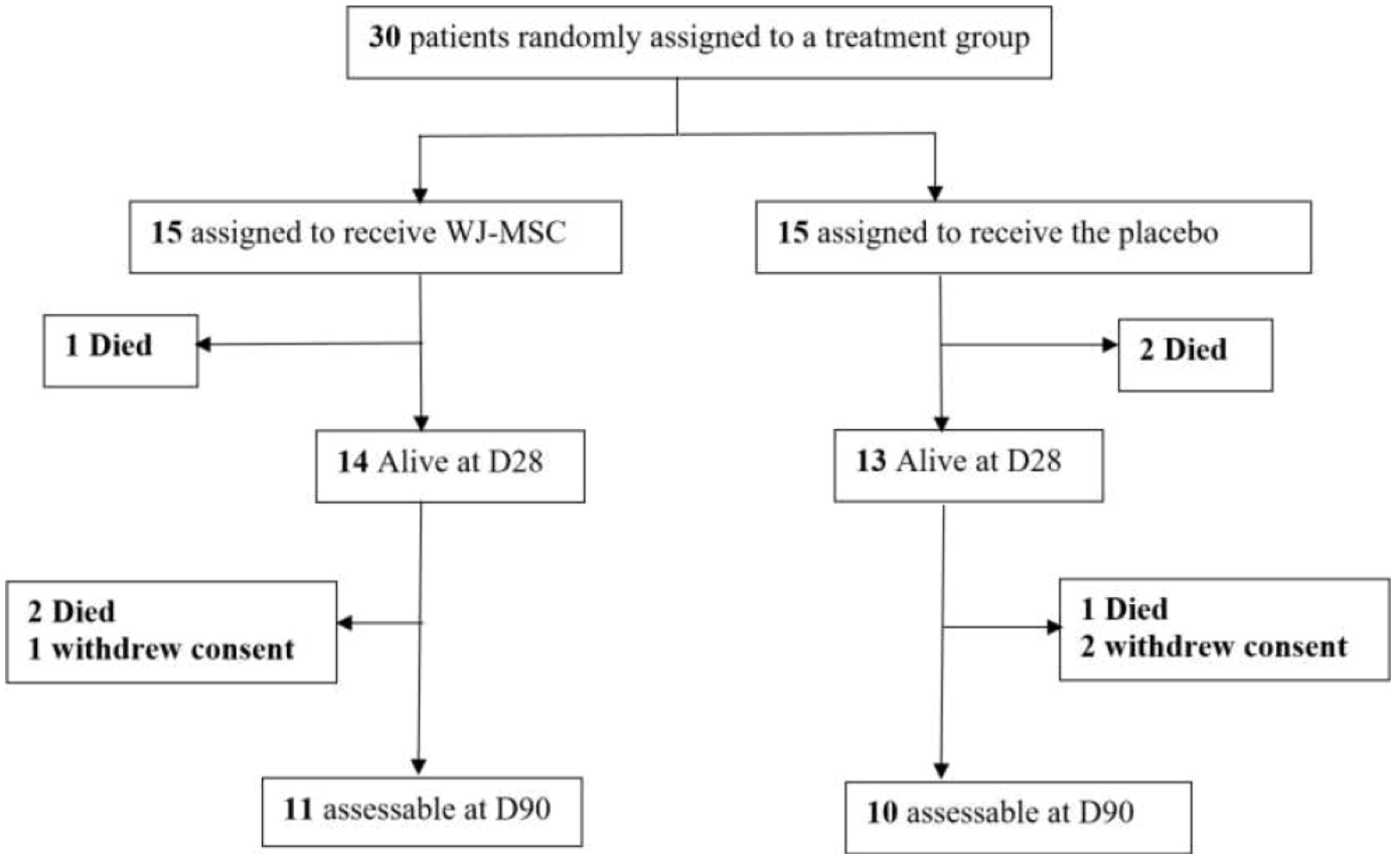
Flow chart of the study.

From November 2020 to May 2021, 30 patients (15 in each group) were randomized in 2 medical ICUs. All of them received the three scheduled infusions except one who was dosed only once. Three patients declined further participation after day 28 (Figure 1).

The median age was 61y (49–66) in the MSC group and 66y (61–70) in the control group. There were more males (87%) in the MSC group than in the control group (47%, *p* = 0.02). Co-morbidities and organ support were similar between groups (Table 1). The SAPS II score was higher in the controls (*p* = 0.04).

**Table 1:** Baseline characteristics.

	WJ-MSC (*n* = 15)	Placebo (*n* = 15)	*p*-values
Age, years (median, IQR)	61 (49; 66)	66 (61; 70)	0.16
Male sex, *n* (%)	13 (87%)	7 (47%)	0.02
Body mass index (median, IQR)	30 (27; 35)	34 (32; 36)	0.19
Symptoms duration days (median, IQR)	9 (5; 11)	7 (4; 13)	0.63
SAPS II (median, IQR)	31 (25; 42)	41 (34; 47)	0.04
SOFA score (median, IQR)	4 (3; 6)	5 (3; 8)	0.4
*Co-morbidities*			
Chronic heart failure	0 (0%)	1 (7%)	1
Hypertension	5 (33%)	10 (67%)	0.14
Diabetes	3 (20%)	4 (27%)	1
Invasive mechanical ventilation	15 (100%)	15 (100%)	1
Prone positioning	7 (47%)	7 (47%)	1
PaO_2_/FiO_2_ (mean, SD)	138 (49)	137 (36)	0.74
PEEP, cmH_2_O (median, IQR)	13 (12; 15)	12.5 (10; 14)	0.44
Compliance, mL/cmH_2_O (median, IQR)	38 (32; 50)	33 (27; 39)	0.27
Corticosteroids, *n* (%)	6 (40%)	9 (60%)	0.47
Vasopressors, *n* (%)	6 (40%)	7 (47%)	1
Renal replacement therapy, *n* (%)	0 (0%)	0 (0%)	1

### Clinical outcomes

**Figure 2:**
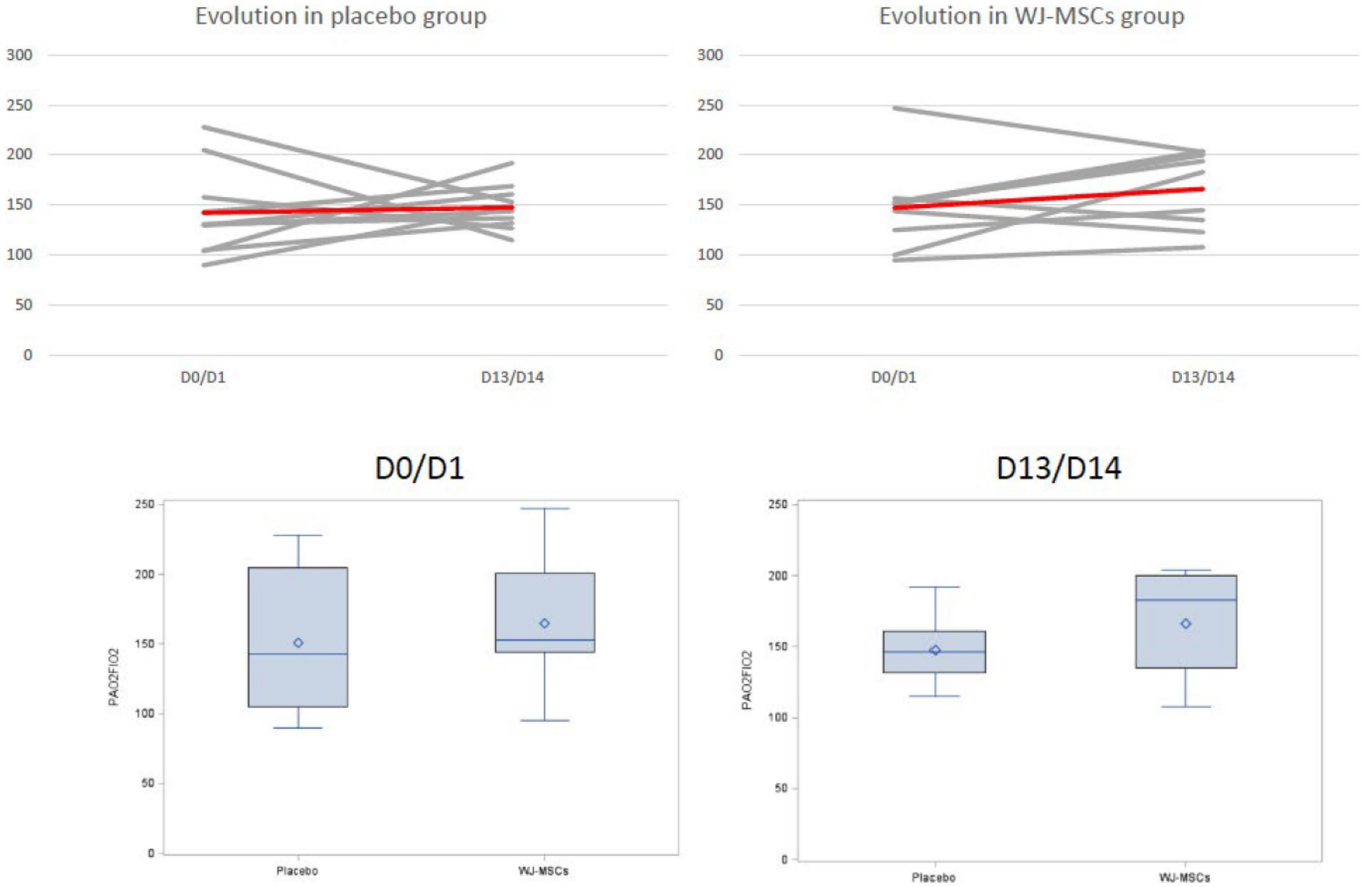
Evolution of the PaO_2_/FiO_2_ ratio in the placebo (left side) and WJ-MSCs group (right side). Lower panels: box plots with medians (horizontal lines) and interquartile range. Baseline arterial blood gases were evaluated just before the first MSC or placebo infusion.

The primary endpoint was the proportion of patients with a PaO_2_/FiO_2_ ratio >200 mmHg at day 10. This outcome was achieved in 18 and 15% of patients from the MSC and control groups, respectively, (*p* = 1) (Table 2 and Figure 2).

**Table 2:** Clinical outcomes.

	WJ-MSC (*n* = 15)	Placebo (*n* = 15)	*p*-values	Effect size (95% CI)
PaO_2_/FiO_2_ day 10 >200 mmHg imputed^a^, *n* (%)	5 (33)	4 (27)	1	0.15 (−0.57; 0.86)
PaO_2_/FiO_2_ day 10 >200 mmHg not imputed (*N* = 25), *n* (%)	2 (18)	2 (15)	1	0.07 (−0.72; 0.88)
PaO_2_/FiO_2_ change (day 14 day 0)	42 (−19; 81)	−2 (−13; 53)	0.39	0.37 (−0.38; 1.12)
Duration of invasive mechanical ventilation, days^b^	12 (5.5; 20.5)	23.5 (10.5; 41.5)	0.15	−0.52 (−1.37; 0.33)
Ventilator-free days at day 28, days	11 (0; 22)	0 (0; 18)	0.2	0.52 (−0.23; 1.28)
Vasopressor-free days at day 28, days	26 (20; 28)	25 (21; 28)	0.88	0.05 (−0.70; 0.79)
Days alive and free of organ support at day 28, days	11 (0; 22)	0 (0; 18)	0.2	0.52 (−0.23; 1.28)
SOFA change (day 14 day 0)	−1 (−2; 0)	−2.5 (−4; −0.5)	0.2	0.64 (−0.20; 1.48)
ICU Length of stay, days	19 (12; 30)	23 (13; 42)	0.34	−0.36 (−1.11; 0.39)
Day 90 mortality, *n* (%)	3 (20)	3 (20)	1	0
Respiratory morbidity at day 90, *n* (%)	13 (87)	12 (86)	1	0.03 (−0.70; 0.76)
Severity of respiratory morbidity (*n* = 25) (%)				
None/moderate	4 (30)	2 (16)	0.61	0.64 (−0.43; 1.72)
Severe	4 (31)	2 (17)		
Very severe	5 (39)	8 (67)		

Data are expressed as median (IQR) or numbers and percentages.

a Last observed value carried forward for 5 missing data at D10.

b In survivors only (*n* = 24).

There was a non-significant trend toward a higher number of ventilator-free days at day 28 in the MSC group (11 vs. 0 days, *p* = 0.2). Vasopressor and extra-renal support did not differ.

ICU length of stay was higher in the control group (23 versus 19 days) though without statistical significance (*p* = 0.34).

**Figure 3:**
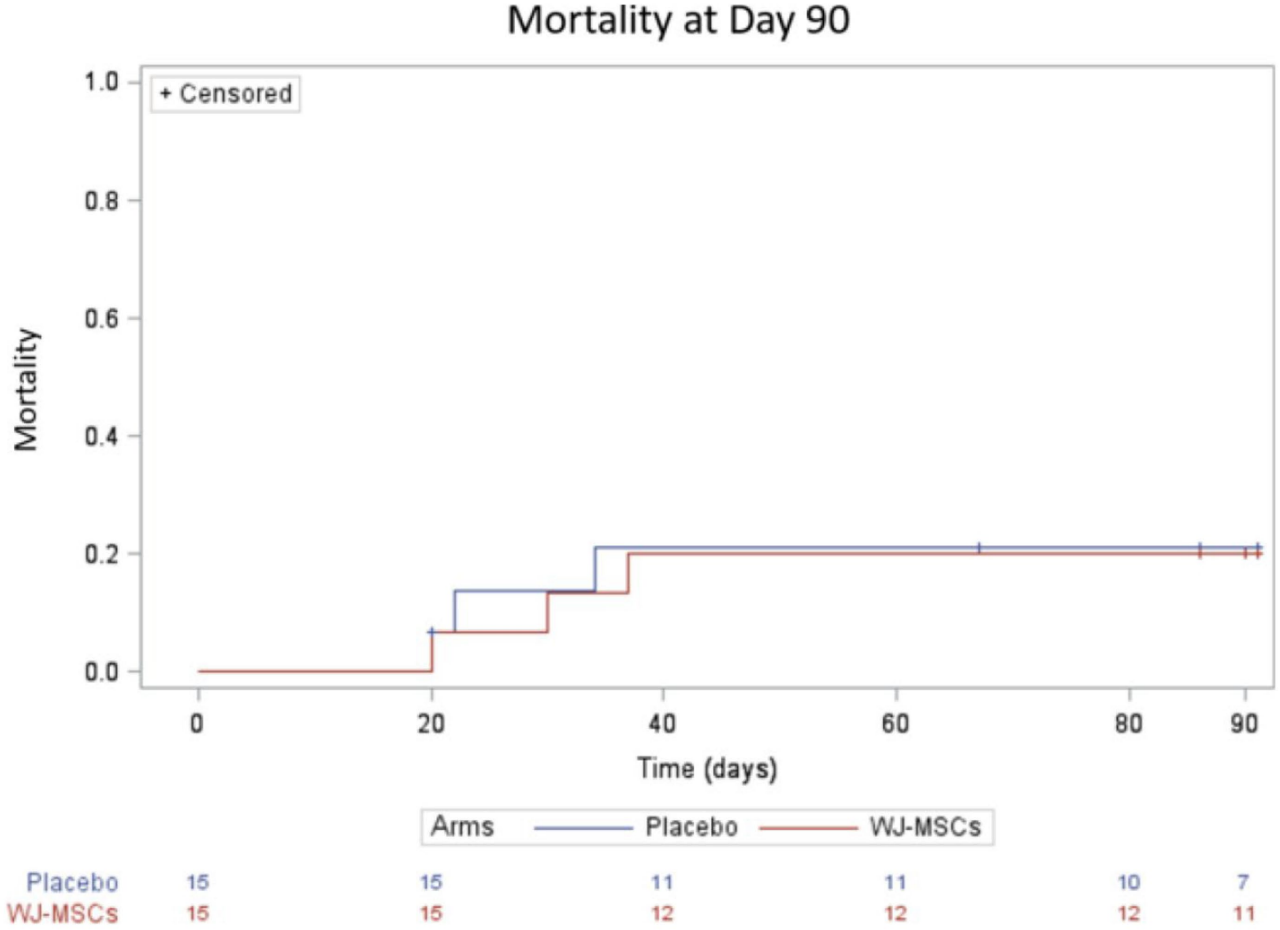
Survival curve up to 90 days.

Mortality did not differ between groups either on day 28 (10%) or on day 90 (20%) (Table 2 and Figure 3).

At day 90, respiratory morbidity could be evaluated in 25 patients without significant differences between groups (Table 2).

### Exploratory biomarkers

There were no differences of Angiopoietin 2 and sRAGE concentrations between groups in terms of baseline values or changes over time (Table 3).

**Table 3:** Exploratory biomarkers.

	WJ-MSC (*n* = 15)	Placebo (*n* = 15)	*p*-values
Angiopoietin 2 “pre”, pg/mL	1,472 (1,032; 2,708)	2,160 (1,286; 3,968)	0.26
Angiopoietin 2 “post”, pg/mL	2,267 (1,217; 4,953)	2,657 (1815; 3,884)	0.78
Angiopoietin 2 change (post-pre), pg/mL	914 (183; 3,037)	105 (−715; 1,471)	0.09
sRAGE “pre”, pg/mL	4,174 (2,431; 5,398)	6,452 (2,727; 14,416)	0.22
sRAGE “post”, pg/mL	1,011 (764; 1,242)	983 (428; 1,214)	0.98
sRAGE change (post-pre), pg/mL	−2,656 (−3,761; −1,599)	−5,469 (−9,859; −1801)	0.13
RT-PCR SARS-CoV-2 day 7 positivity (*N* = 28)	76.90%	53.30%	0.25
RT-PCR SARS-CoV-2 day 14 positivity (*N* = 21)	62.50%	46.20%	0.66
RT-PCR SARS-CoV-2 day 21 positivity (*N* = 15)	42.90%	50.00%	1

“Pre”, just before the first infusion; “Post”, the day after the last infusion; RT-PCR, reverse transcriptase-polymerase chain reaction; s-RAGE, soluble receptor for advanced glycation end products.

The proportion of patients that remained positive for SARS-CoV-2 on days 7, 14, and 21 did not differ between groups.

**Figure 4:**
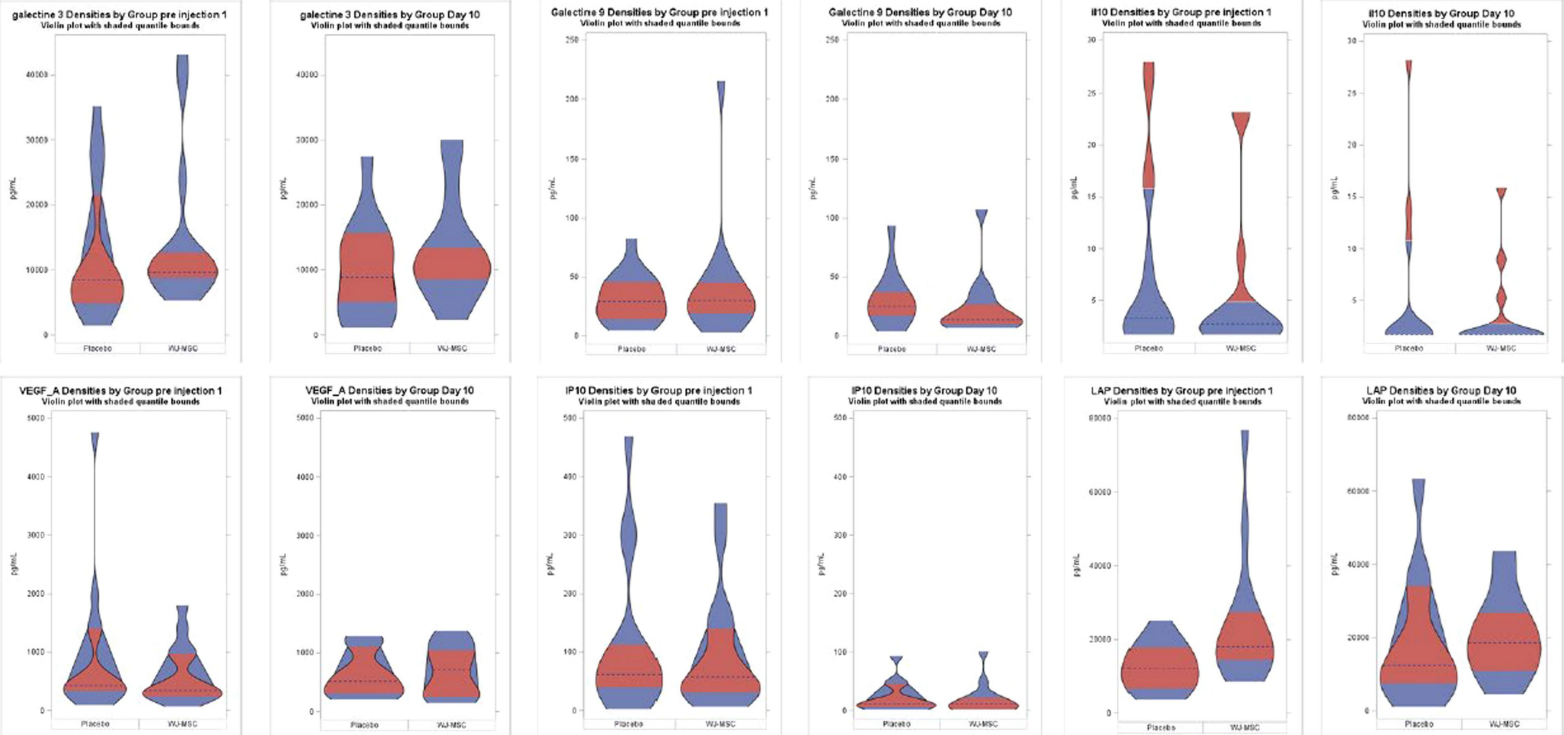
Plasma cytokines concentrations. Values are expressed in pg./mL. Horizontal dashed lines represent the medians; in red, the 25th–75th percentiles range.

We also found no difference in the plasma concentrations of Galectin-3, Galectin-9, VEGF, IL-10, IP10, and LAP at baseline or day 10 between the MSC and control groups (Figure 4). Concentrations of IFN-γ, TNF-α, IL-1α, and IL-1β remained barely detectable in every patient at both times.

Regarding lymphocyte count, we observed no differences in CD4^+^, CD8^+^, NK, MDSC, and Tregs cells both at baseline and over time (not shown).

Cytokines plasma concentrations (baseline or change over time) were similar between patients with favorable (alive and duration of invasive mechanical ventilation <14 days) and unfavorable outcomes (death or invasive mechanical ventilation >14 days) (not shown). By contrast, we found that patients with a favorable evolution had an increase in CD4^+^, CD8^+^, NK, T cells, and Tregs cells (Supplementary Figure S1).

### Safety analysis

There was no between-group difference in terms of adverse or serious adverse events frequency (Table 4). In one patient, death was reported by the clinician to be possibly related to infusion but this was eventually ruled out by the DSMB.

**Table 4:** Adverse events.

	WJ-MSC	Placebo	Total	*p*-value*
**Adverse events (total)**				
Subjects with AE	14/15 (93.3%)	14/15 (93.3%)	28/30 (93.3%)	1
AEs reported	149/294 (50.7%)	145/294 (49.3%)	294 (100%)	0.82
Subjects with SAE	5/15 (33.3%)	3/15 (20.0%)	8/30 (100%)	0.68
SAEs reported	8/149 (5.4%)	12/145 (8.3%)	20/294 (6.8%)	0.52
AEs by severity				0.46
Mild	82/149 (55.0%)	59/145 (40.7%)	141/294 (48%)	
Moderate	49/149 (32.9%)	70/145 (48.3%)	119/294 (40.5%)	
Severe	18/149 (12.1%)	16/145 (11.0%)	34/294 (11.6%)	
AEs by treatment relatedness				0.85
Possibly related	3/149 (2.0%)	6/145 (4.1%)	9/294 (3.1%)	
Death	3/15 (20%)	3/15 (20%)	6/30 (20.0%)	1
**Adverse events D0 to D14**				
Subjects with AE	14/15 (93.3%)	14/15 (93.3%)	28/30 (93.3%)	1
AEs reported	107/198 (54.0%)	91/198 (46%)	198 (100%)	0.26
Subjects with SAE	2/15 (13.3%)	1/15 (6.7%)	3/30 (10.0%)	1
SAEs reported	3/107 (2.8%)	5/91 (5.5%)	8 (100%)	0.99
AEs by severity				0.65
Mild	59/107 (55.1%)	45/91 (49.5%)	104/198 (52.5%)	
Moderate	40/107 (37.4%)	42/91 (46.2%)	82/198 (41.4%)	
Severe	8/107(7.5%)	4/91 (4.4%)	12/198 (6.1%)	
**AEs by treatment relatedness**				
Possibly related	3/107 (2.8%)	6/91 (6.6%)	9/198 (4.5%)	0.99
Death	0/15 (0%)	0/15 (0%)	1/30 (3.3%)	1
**Adverse events after D14**				
Subjects with AE	8/15 (53.3%)	11/15 (73.3%)	19/30 (63.3%)	0.26
AEs reported	35/81 (43.2%)	46/81 (53.8%)	81 (100%)	0.22
Subjects with SAE	3/15 (20%)	4/15 (26.7%)	7/30 (23.3%)	1
SAEs reported	5/35 (14.3%)	7/46 (15.2%)	12/81 (14.8%)	0.86
AEs by severity				<0.001
Mild	20/35 (57.1%)	9/46 (19.6%)	29/81 (35.8%)	
Moderate	5/35 (14.3%)	25/46 (54.3%)	30/81 (37%)	
Severe	10/35 (28.6%)	12/46 (26.1%)	22/81 (27.2%)	
**AEs by treatment relatedness**				
Possibly related	0/35 (0%)	0/46 (0%)	0/81 (0%)	NA
Death	3/15 (20%)	3/15 (20%)	5/30 (16.7%)	1

D-dimer concentrations did not differ between groups on day 1 (2,737 ± 2,735 vs. 2,757 ± 2,803 ng/mL) or on day 10 (3,194 ± 2,607 vs. 3,635 ± 2,165 ng/mL). Seven thromboembolic events occurred (4 versus 3 in the control group), none were reasonably related to the experimental product.

In the MSC group, two out of 15 analyzable patients had pre-formed DSA before any cell injection: one had an anti-HLA-B44 and anti-HLA-Cw12 with MFI of 1875 and 13,614, respectively. The other patient had an anti-HLA-DQ7 (MFI 2270) and anti-HLA-DRB3*02:02 (MFI 1652).

At day 28, none of the six analyzable patients exhibited DSA.

At day 90, two out of 10 analyzable patients had DSA: one patient developed a *de novo* anti-HLA-B44 with a MFI of 1,575, although this specificity was negative ay days 0 and 28. It is worth noting that this patient had received blood product transfusions with clinical allergic reaction after day 28. The second patient exhibited the preformed anti-HLA-DQ7 and anti-HLA-DRB3*02-02 with increased MFI compared to day 0 (3,029 and 2,402 respectively) plus a *de novo* DSA towards HLA-DRB4*01:03 with a MFI slightly above the cut-off (1,068 compared to 708 at day 0).

## Discussion

This study demonstrates the feasibility and tolerability of 3 infusions of WJ-MSCs in patients with acute respiratory distress syndrome secondary to SARS-Cov-2 but fails to meet the primary objective of demonstrating a greater number of patients with a PaO_2_/FiO_2_ > 200 mmHg at 10 days.

However, it shows a trend toward a reduction in the number of days under invasive mechanical ventilation and the ICU length of stay.

Several teams have reported variable results after single or repeated infusions of umbilical cord MSC in patients with COVID-19-related respiratory distress. These clinical trials were based on the described effects of MSCs in immunomodulation, and their putative impact in ARDS related to other agents (15–17), as well as on the pathophysiology of SARS-CoV-2-induced ARDS involving the activation of the inflammasome, and the synthesis of pro-inflammatory cytokines (18, 19).

Lanzoni et al. reported a survival improvement in 24 COVID-19 ARDS patients treated with two infusions of 100 × 10^6^ MSCs at days 0 and 3: 91% in the MSC group vs. 42% for the control group (20). Similar results were obtained by Dilogo et al. in 40 patients (21), and by Häberle et al. in 18 patients (22). Zhu et al. reported a shorter hospital stay and faster symptom resolution in 29 patients who received one infusion of 1 × 10^6^/kg umbilical cord MSCs, compared with 29 controls (23). Shu et al. reported better survival for 12 patients who received an infusion of 2 × 10^6^/kg umbilical cord MSCs compared with 29 controls (24). Other teams have confirmed the feasibility of the injection of umbilical cord MSCs in patients with COVID19 infection (25, 26).

On the other hand, Monsel et al. did not demonstrate the benefit of three infusions of umbilical cord MSC, while confirming their good tolerance (27). The same conclusions were made in the randomized trial of Bowdish et al. on 223 patients, with two infusions of 2 × 10^6^/kg MSC (Remestemcel-L), which showed comparable survival at day 30, as well as similar mechanical ventilation-free days at day 60 (28). The same finding was recently published by a team from United Kingdom of Great Britain and Northern Ireland in a randomised double-blind trial involving 60 patients with moderate-to-severe COVID-related ARDS, 30 of whom received an intravenous injection of 400 million CD362-enriched umbilical cord-derived MSCs versus placebo (29). A striking aspect of the four positive studies (20–23) is a very high mortality rate in the control group, as compared to the negative ones including our trial in which the survival rate was 80%.

We did not find any difference in terms of T-cells, NK cells, Tregs, myeloid-derived suppressive cells (MDSC) counts, or in the concentration of cytokines and other molecules between groups, in either baseline levels or kinetics.

Cytokines plasma concentrations remained low in our patients, in contrast to what was described in Lanzoni’s study (20). This low level of inflammatory molecules in our study could be related to the early use of corticosteroids (in 50% of patients) or tocilizumab (in 17% of patients). Hyper, as well as hypo-inflammatory phenotypes, have been described in COVID-19 ARDS patients, and such variability should be considered when dealing with immuno-modulatory therapies (30). Apart from corticosteroids, other therapies such as tocilizumab or antivirals could have an impact on the cytokine environment and thus on the effect of MSC. However, in the negative trial reported by Bowdish (28), more than 80% of patients had received corticosteroids, and 2/3 of them remdesivir, whereas in the positive Lanzoni’s trial (20), 83% of the patients had also received corticosteroids, and the majority (75% in the MSC group and 58% in the control group) were concomitantly receiving remdesivir.

Other variables also render the comparison of the studies hazardous:

First, the viral strain, which has evolved from the start of the pandemic, may alter the impact of the virus on the inflammatory response. However, the trials mentioned above were conducted between the spring of 2020 and the end of 2020. If the period is similar, the geographical areas differ (United States, Germany, France, Indonesia, China), and therefore possibly the viral strain.

Second, the origin of MSCs and their manufacturing (culture medium, number of passages, number of donors) differs: bone marrow MSCs for the Häberle and Bowdish studies, umbilical cord MSCs for the other four. The dosage and the number of administrations (from 1 to 3) also vary greatly.

Third, the severity of ARDS is also variable: all patients receiving invasive mechanical ventilation in the Bowdish’s study (28) and our own, while more than half of the patients were not intubated in the Lanzoni’s trial (20). It is plausible that lung injury was already too severe in invasively ventilated patients to allow for a benefit of MSC administration.

Our study showed a good safety profile and a very low risk of alloimmunization following iterative WJ-MSC infusions. This was in line with the findings of Monsel et al., with three patients out of 21 who received at least one WJ-MSC infusion developing donor-specific antibodies against the HLA molecules of the MSCs at D14, though at a low level (23). To our knowledge, this has not been reported after infusions of Obnitix MSCs, which were derived from eight different bone marrow donors (31).

Several limitations of this study deserve to be discussed. (i) The major one stems from the low number of included patients: we calculated the sample size based on an estimation of the frequency of patients with a PaO_2_/FiO_2_ > 200 mmHg at day 10 of 75% in the MSC group and 25% in the control group. This estimate derived from the results from the first study in the field (32). This was inadequate as less than 20% of patients reached this PaO_2_/FiO_2_ ratio in both groups. Although the study was underpowered, the lack of any difference in the 2 groups argues against an effect of the MSCs infusion on this parameter. (ii) There was a slight imbalance at randomization regarding gender and severity scores favoring the experimental group. (iii) We only included moderate to severe ARDS patients receiving invasive mechanical ventilation and thus could not elaborate on the MSC effect in a less severely ill population. (iv) Finally, we chose to administer a three doses regimen within 5 days: whether this administration scheme had been optimal is also unknown.

## Conclusion

WJ-MSC administration to COVID-19-related moderate to severe ARDS patients was safe and well tolerated. However, MSCs were not significantly associated with any clinical or biological improvements.

## Data availability statement

The raw data supporting the conclusions of this article will be made available by the authors, without undue reservation.

## Ethics statement

The studies involving human participants were reviewed and approved by Comité de Protection des Personnes Sud Mediterranee III, reference: EudraCT 2020-002772-12, acceptance on 17 November 2020. The patients/participants provided their written informed consent to participate in this study.

## Author contributions

CP, CL, DB, and SG designed the study and wrote the first draft of the manuscript. AK, J-FC, and SV included patients. CL, LR, MG, VD, and DB prepared cells and performed quality controls. AD, MC, MP, and AA performed biological analyses. NP performed safety analyses. HR performed statistical analyses.

## Funding

This trial was funded by the Programme Hospitalier de Recherche Clinique supported by the French Ministry of Health.

## Conflict of interest

DB is a co-founder and member of the scientific advisory board of StemInov, a French Stem Cell company. She holds patent on the use of WJ-MSCs for sepsis licensed to StemInov.

The remaining authors declare that the research was conducted in the absence of any commercial or financial relationships that could be construed as a potential conflict of interest.

## Publisher’s note

All claims expressed in this article are solely those of the authors and do not necessarily represent those of their affiliated organizations, or those of the publisher, the editors and the reviewers. Any product that may be evaluated in this article, or claim that may be made by its manufacturer, is not guaranteed or endorsed by the publisher.

## Footnotes

^1^ https://clinicaltrials.gov/ct2/show/NCT04625738

## Abbreviations

ARDS: Acute respiratory distress syndrome
IL: Interleukin
MSC: Mesenchymal stem cells
SOFA: Sequential organ failure assessment
WJ: Wharton jelly
