# Supplementary material for: Efficacy of Wharton Jelly Mesenchymal Stromal Cells infusions in moderate to severe SARS-Cov-2 related acute respiratory distress syndrome: a phase 2a double-blind randomized controlled trial
Source: Front Med (Lausanne). 2023 Aug 29;10:1224865. doi: 10.3389/fmed.2023.1224865 (PMC10495568; doi:10.3389/fmed.2023.1224865)

Supplementary data

<u>WJ-MSCs production</u>

MSCs were produced from Wharton jelly of umbilical cord by explant method. Umbilical cords were collected at the Maternity of the Nancy University Hospital. Before collection, pregnant mothers signed an informed consent in compliance with French national legislation regarding human sample collection, manipulation, and personal data protection. This collection was approved by the Nancy Hospital ethics committee and the French ministry of research.

Upon receipt, the umbilical cord was immersed in an antibiotic bath (gentamicin, amoxicillin, vancomycin and amphotericin B) for one hour and then cut into 5 cm pieces. These pieces were placed in culture dishes (Dutscher, France) for adherence without medium during 15 minutes. Afterwards, medium was added (alpha MEM 5% Platelet Lysate 30i (Macopharma)) and cells were incubated in hypoxia (5% of O2 and 5% of CO2). After 10 days, the cord pieces were removed and the medium renewed. When the cells reached 80% confluence, they were detached by trypsin action (Fischer Scientific, France) and seeded in passage 1 (P1) in cellstacks (Macopharma, France) at a density of 1000 cells/cm^2. MSC were cultured until passage 2 and then frozen in a solution composed of 80% albumin and 10% Dimethyl sulfoxide (DMSO) at a minimum concentration of 1.10^6 MSC/ml.

MSCs in passage 2 were thawed in a water bath at 40 °C, washed and seeded in P3 at a density of 1000 cells/cm^2. After 80% confluence, MSCs were detached by trypsin action, washed and frozen in a solution composed of 80% albumin and 10% Dimethyl sulfoxide (DMSO) at a minimum concentration of 1.10^6 MSC/ml. MSCs were stored in nitrogen vapor until use. Four umbilical cords were used to produce 10 batches of MSCs in P3.

After randomization, 1 to 4 MSCs bags were thawed in a water bath at 40°C and washed. MSC concentration was adjusted to the patient's weight. All MSCs administered to a patient came from a single umbilical cord.

WJ-MSCs quality controls

Infectious markers (HIV, HCV, HBV, HTLV, EBV, CMV, Syphilis, Toxoplasmosis, Sars-Cov2) of umbilical cord donors were determined in the virology laboratory of Nancy hospital.MSCs were labeled with the anti-CD90, CD73, CD44, CD105, CD34, CD45, CD11b, CD19, HLA-DR mAbs (Stemflow hMSC Analysis kit, Becton Dickinson, USA) to determine phenotype. Microbiology was tested on culture supernatant and final product by aerobic and anaerobic bactec plus (Becton Dickinson). Cell count was performed by flow cytometry and viability evaluated by 7AAD labeling (MACSQuant, Miltenyi). Clonogenicity percentage was determined by a CFU-F assay. Karyotype was performed by genetic laboratory of Nancy hospital on twenty mitoses and Telomerase activity by qRT-PCR in cytogenetics laboratory of Clermont-Ferrand hospital. MSC immunogenicity and immunomodulation were evaluated by lymphocyte proliferation assay (DELFIA® Cell Proliferation kit) like previously described .

Supplementary Table 1: MSC quality controls

	Specifications	Collection	Master cell bank: Enf of P0 (n=4)	Master cell bank: End of P1 (n=4)	Master cell bank: End of P2 (n=4)	Working cell bank: Thawing P2 (n=10)	Working cell bank: End of P3 (n=10)
Infectious Markers	Negative	Negative					
Microbiology	Negative	Negative	Negative	Negative	Negative	Negative	Negative
Viability	≥ 80% cell bank ≥ 50% final product		94.3 (+/-1.8) %	92.7 (+/- 8.4) %	95.7 (+/- 4.9)%	90.9 (+/- 5.4)%	94.3 (+/-1.8) %
Phenotype : CD73; CD90; CD105; CD44	≥ 70%		97.4 (+/- 1.9) %	99.6 (+/- 0.4)%	99.0 (+/- 1.2) %		99.4 (+/- 1.0) %
Phenotype : CD45; CD34; HLA-DR	≤ 5 %		0.45(+/-0,2) %	0.7 (+/- 1.3)%	0.3 (+/- 0.4) %		0.20 (+/ -0.2) %
CFU-F	≥ 0.1%		23.0 (+/- 5.8) %	27.0 (+/- 9.9)%	23.4 (+/- 22.9) %		15.5 (+/- 6.4) %
Mixed lymphocyte reaction : % Inhibition	≥ 20%						31.6 (+/-28.2) %
Mixed lymphocyt reaction : % Imunogenicity	≤ 10%						5.1 (+/-4.4) %
Karyotype	46 XX or 46 XY						46 XX or 46 XY
htert	Negative						Negative
Yields after thawing	/					81.6 (+/- 10.6) %	

SUPPLEMENTARY FIGURE 1

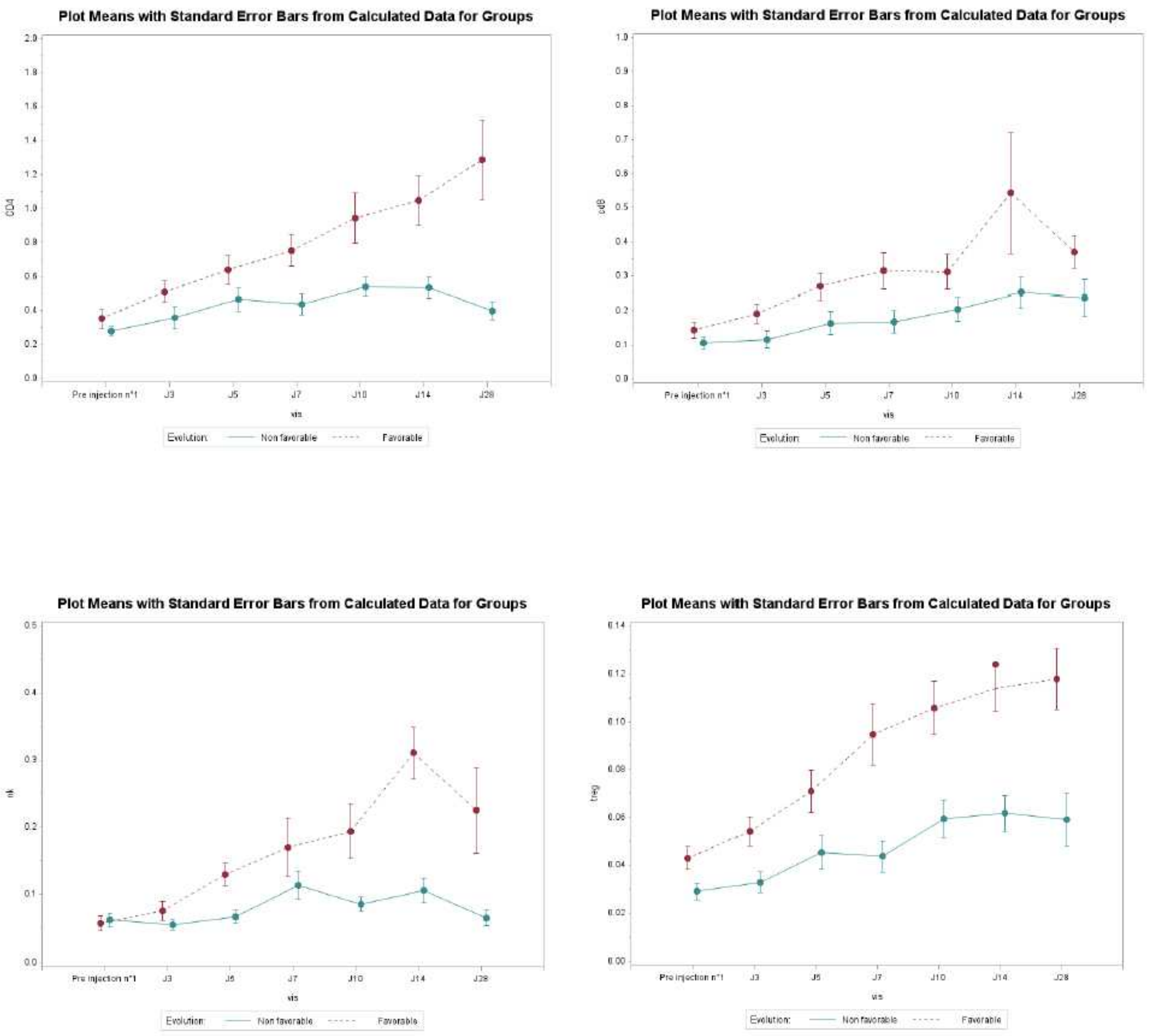

Supplement: Supplementary file 1 [file Presentation_1.pdf]
